# The Effects of Transcranial Direct Current Stimulation of Dorsolateral Prefrontal Cortex on Reduction of Craving in Daily and Social Smokers

**Published:** 2019-10

**Authors:** Nader Hajloo, Asghar Pouresmali, Jaber Alizadeh Goradel, Mehri Mowlaie

**Affiliations:** 1 Department of Psychology, University of Mohaghegh Ardabili, Ardabil, Iran.; 2 Young Researchers and Elite Club, Ardabil Branch, Islamic Azad University, Ardabil, Iran.; 3 Department of Psychology, University of Shahid Beheshti, Tehran, Iran.

**Keywords:** *Smokers*, *Transcranial Direct Current Stimulation (tDCS)*

## Abstract

**Objective:** Tobacco smoking is an addictive behavior with many psychological side effects, and many smokers are unable to quit it. Despite various treatments for smoking cessation, there is an urgent need to develop and utilize a noninvasive technique with high efficacy. This study aimed to determine the effects of transcranial direct current stimulation (tDCS) of dorsolateral prefrontal cortex (DLPFC) on reduction of craving in daily and social smokers.

**Method** **:** This pretest-posttest quasi-experimental study, in which the participants were randomly assigned into sham and active groups, was performed on 40 daily and social smokers. Stimulation was delivered over the left DLPFC at a 2 m/A during 10 twenty-minute sessions for 5 weeks. The participants filled out the Desire for Drug questionnaire (DDQ) before and after intervention. Analysis of covariance was used for data analysis.

**Results: **A significant decrease was found in the number of cigarette smoking in both daily and social smokers compared to the sham group. Moreover, the results indicated that anodal tDCS on F3 and Cathodal tDCS on F4 has significant effects on nicotine craving (P < 0/000).

**Conclusion: **The results of the present study showed that the current tDCS of DLPFC decreases the craving of smoking. This noninvasive brain stimulation technique targeted at DLPFC area may be a promising method for reducing and treating smoking craving.

Tobacco smoking is one of the most serious health problems worldwide (1) and is the third most frequent cause of mortality in industrialized countries (2). Tobacco-related diseases are estimated to cause more than 5 million deaths in the developed world annually (3). By 2030, the yearly smoking-related death is expected to rise to 8 million (3).

Tobacco addiction is characterized by the loss of control over cigarette smoking and the compulsive smoking behavior regardless of the negative consequences (4). Nicotine is the main psychoactive ingredient in tobacco smoking and is believed to result in its addictive behaviors (5). Nicotine releases neurotransmitters, such as dopamine (DA), noradrenaline (NE), serotonin, endogenous opioids, Y-aminobutyric acid (GABA), and glutamate (6). 

Initiation of addiction is due to nicotine’s ability to release DA in nucleus accumbens (NAc) (7). In nicotine dependency, relapse rate is estimated to be 85% for counseling therapy alone and 78% for counseling combined with medication (8). Many brain regions are involved in smoking addiction (eg, mesocorticolimbic system, insula cortex, prefrontal cortex, and hippocampus), and the manipulation of the activity of these brain regions can show a modification of smoking behavior (9, 10, 11). Despite the involvement of some brain regions in smoking, this phenomenon is associated with adverse physical, psychological, and social effects and causes lung cancer, cardiovascular diseases (CVDs), and chronic respiratory diseases and remains the leading cause of death (2).

Several strategies are used to prevent and control tobacco consumption worldwide such as smoke-free legislations and prevention campaigns. Despite existing approaches to control smoking, including cognitive-behavioral therapies, nicotine-replacement therapies (NRT), pharmaceutical treatments (bupropion and varenicline) (12), and combination of these therapies (13), relapse to smoking is highly prevalent. Thus, new brain stimulation techniques are needed to help smokers who want to quit smoking (14). Transcranial direct current stimulation (tDCS) is one of the new noninvasive methods that has been widely used in psychological disorders and changing brain activity (13, 14).

Many studies have confirmed that tDCS can modulate cortical excitability (15, 16). Anodal stimulation increases and cathodal stimulation decreases excitability (15). In other words, anodal stimulation induces neural depolarization firing, while cathodal stimulation induces hyperpolarization (19, 20). Studies have shown that prefrontal cortex, especially DLPFC, is involved in drug craving (21, 22). It has been suggested that anodal tDCS may increase activity in the DLPFC, reinforcing drug-avoidance behavior (23).

Several studies have investigated the effects of tDCS in individuals with tobacco use disorder (TUD) and found that tDCS can reduce craving (24, 25) and smoking (26, 27) when applied over either the left or right DLPFC; also, they found that repeated sessions of tDCS can have a cumulative effect on smoking behavior (25). The sessions of using tDCS in these studies were different from 4 to 15; also, some negative findings on craving (27) and smoking (28) have been reported. In one study, researchers failed to find a significant effect of a single session of tDCS applied with the anode placed over the left DLPFC and the cathode over the right supraorbital region on craving (27). In another study, a significant increase was found in latency to smoke and a significant decrease was observed in the total number of cigarettes smoked 1 hour after a single tDCS session, applied with the same electrode montage; however, no effect on self-reported number of cigarettes smoked was found in the following 24 hours (28). One of the reasons of these contradictory results can be the number of sessions. Furthermore, they did not use objective measures for craving. Also, few investigations have been conducted on daily and social smokers to address these limitations. The present study was conducted to investigate the effects of 10 sessions of tDCS on smoking craving in daily and social smokers; moreover, this was the first study to compare the effectiveness of tDCS on daily and social smokers, in line with previous studies (29).

## Materials and Methods

***Study Design & Participants***

This was a quasi-experimental study with pretest, posttest, and follow-up assessments with control group. Quasi-experimental design was chosen because group selection was available. A total of 40 college students (40 males, aged 18-30 years), without a self-reported history of mental or neurological disorder and any other substance abuse, volunteered to participate in this double-blind experiment. None of the participants knew into which groups they would be placed. They were randomly assigned either into active or sham groups and took part in 10 sessions of tDCS at the Beautiful Mind Counseling Center 2 times a week for 5 weeks in 2015.

***Inclusion & Exclusion Criteria***

Inclusion Criteria were as follow: (1) age 18-30 years; (2) no previous mental disorder; (3) willingness to provide written informed consent; (4) willingness to participate in a long-term follow-up study (1 month); (5) no brain injury; (6) at least 2 years smoking history with more than 10 cigarettes daily for daily smokers and 5 cigarettes for social smokers; and (7) no smoking cessation therapy history. The exclusion criteria were as follow: (1) history of alcohol and substance abuse; (2) history of severe neurological disorders; and (3) uncontrolled medical problems. Daily smokers were defined as those who smoke within 1 hour of waking up and smoke more than 10 cigarettes a day (approximately 106 cigarettes per week).

***Sampling***

Participants were selected from Mohaghegh Ardabili University. They were randomly divided into 2 groups of daily and social smokers, based on the amount and frequency of consumption. Daily smokers were defined as those who smoke within 1 hour of awakening and smoke more than 10 cigarettes per day (approximately 106 cigarettes per week). Social smokers were defined as those smoking at intermittent times and no more than 20 cigarettes per week (approximately 12 cigarettes per week); also, they were randomly assigned into active and sham groups.

***Intervention Protocol***

In this study, tDCS was performed with two saline-soaked sponge electrodes applied over the participants' scalps. Electrodes were placed according to the international 10-20 EEG systems. Active tDCS consisted of delivering a constant current of 2 m/A for 20 minutes (ramp up/down: 30 seconds). For the sham tDCS, they undergone the same procedure but no current was applied. Each participant received a total of 10 sessions of tDCS 2 times a week with 72 hours interval between sessions. Anodal tDCS was placed on left DLPFC (F3) and Cathodal tDCS on right DLPFC (F4). DDQ questionnaire was administered before the stimulation (first session) and after stimulation (last session). Follow-up assessment was one month after the sessions and the DDQ questionnaire was administered again. All the processes were done by authors with tDCS degree.

***Ethics***

All participants were provided with necessary explanations on the purpose of the study, how it was done, side effects, and effects of the treatment, and the researchers answered all the questions. All participants were asked to provide a written informed consent and they were informed they could withdraw from the study at any time. This study was in agreement with the principles of Declaration of Helsinki-Ethical Principles for Medical Research Involving Human Subjects. All stimulation sessions were performed by the same researchers and at the same time of the day.

***Statistical Analysis***

Statistical analysis was performed using SPSS software version 20 (SPSS Inc, Chicago, I1 and USA). Leven test was used to examine the similarity of variances. Also, analysis of covariance was used to compare tDCS effectiveness in 2 active and sham groups.

***Instruments***

1. Desires for Drug Questionnaire (DDQ): This is a 14-item questionnaire developed by Franken, Hendriks, and Van den Brink (30). It is derived from Desire for Alcohol questionnaire (DAQ), which was used to determine dependency to heroin, but due to its ability to assess whole narcotic substance, later, it was used to assess craving of another substance, such as cigarette. This questionnaire has three subscales that measure instant craving: (1) desire and intention; (2) negative reinforcement; and (3) control. Fanken (30) reported the validity of the whole scale to be 0.79 using Cronbach’s alpha. Also, the validity of the subscales of desire and intention, negative reinforcement, and control was found to be 0.77, 0.80, and 0.75, respectively. Suitable validity and reliability of this scale has been reported in Iran (31).

## Results

All participants were male college students. The mean age of daily smokers was 21/83 years and the mean age of social smokers was 21/33. Of the participants, 90% were single. Baseline demographic and clinical characteristics of participants enrolled in active and sham groups are presented in Table 1. There were no statistical differences between active and sham groups for age, duration of smoking, and cigarette consumption at baseline.

The mean scores and standard deviations of the craving (DDQ scores) are shown in Table 2. The mean of DDQ scores at follow-up and posttest was lower than pretests in active group. In sham group, there was no change in DDQ score in 3 assessments.

The effectiveness of tDCSon craving was determined by analysis of covariance. First, equality of the slope of regression line and Levene test were used to examine the similarity of variances as presumes of analysis of covariance were processed. The results revealed no significant interaction between groups with pretest (F 1, 12=3.76, F=1, 12=4.27; p=0<05, P=0<05) in ANCOVA. 

The results of Table 3 show that after modification of pretest scores, significant differences were observed between active and sham groups (p < 0/001, F1, 12 1/371). Therefore, the first hypothesis was confirmed based on the effectiveness of tDCS on reduction of craving in daily smokers.

As seen in table 4, significant differences were found between active and sham groups (p < 0/001, F1, 12 1/103). Therefore, the hypothesis of the effectiveness of tDCS on reduction of craving in social smokers was confirmed.

**Figure 1:**
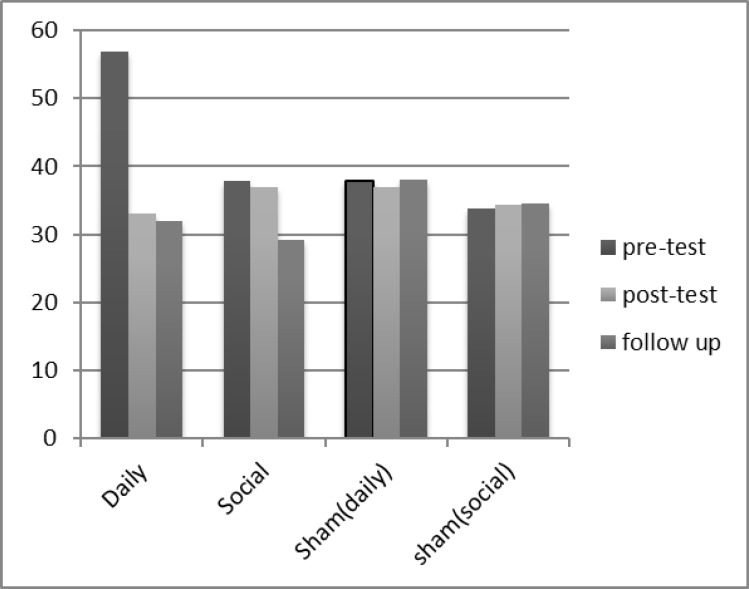
Group Mean Percentage Changes in the Craving Questionnaire Score in Active (Daily and Social Smokers) and Sham Stimulation

Finally, figure 1 shows mean percentage changes of craving scores in active and sham groups. As seen, post-test and follow up scores in active groups has decreased. 

**Table 1:** Baseline Demographic and Clinical Characteristics of Participants Enrolled in Active and Sham Groups

	**Active ** **Group ** **(N=20)** **Mean**	**SD**	**Sham ** **Group ** **(N=20)** **Mean**	**SD**
Age	21.83	0.38	21.33	0.49
Education				
BSc	75	4.5	65	3.9
M.A	25	2.3	35	3.7
Duration of smoking (years)	4	2.2	3	1.3
Cigarette consumption (self-reported)	110	5.6	115	6.9

**Table 2:** Means and Standard Deviations of Craving in Daily and Social Smokers at Pretest, Posttest, and Follow-up

**Groups**	**Pretest (Craving)** **Pre-test**				**Posttest (Craving)** **Post-test**				**Follow-up (Craving)**			
	**Active**		**Sham**		**Active**		**Sham**		**Active**		**Sham**	
	Mean	SD	Mean	SD	Mean	SD	Mean	SD	Mean	SD	Mean	SD
Daily smokers	56.83	20.36	63.66	17.60	33	15.62	63.78	26.40	32	16.78	63.80	26.58
Social smokers	37.83	7.41	33.83	3.06	30	5.62	34.33	2.65	29.23	6.43	34.59	2.78

Note DDQ: Desires for Drug Questionnaire; M=Mean; SD=Standard deviation

**Table 3:** Results of Analysis of Covariance of Craving in Daily Smokers

**Variable**		**Sum of Squares**	**Df**	**Mean Squares**	**F**	**sig**	**Eta Squares**
	Pretest	19521/333	1	19521/333	41/488	0/000	0/80
Craving	Group	645/333	1	645/333	1/371	0/003	0/78
	Errors	4705/333	10	470/533			
	total	24872/000	12				

**Table 4:** Results of Analysis of Covariance of Craving in Social Smokers

**Variable**		**Sum of Squares**	**Df**	**Mean Squares**	**F**	**sig**	**Eta ** **squares**
	Pretest	15265/333	1	15265/333	789/586	0/000	0/98
Craving	Group	21/333	1	21/333	1/103	0/003	0/78
	Errors	193/333	10	19/333			
	total	15480/000	12				

## Discussion

To our knowledge, this was the first sham-controlled study that evaluated the effects of 10 sessions of tDCS on craving of daily and social smokers. In the present study, the results of analysis of covariance indicated significant reduction in desire for drug questionnaire scores in both active stimulations (daily and social smokers) compared to sham stimulation. The results of this study showed that active anode/left, cathode/right DLPFC stimulation reduced nicotine craving significantly as compared to sham stimulation. Moreover, participants consumed a fewer number of cigarettes after both active stimulation conditions and in the follow-up. Also, the results showed that after sham stimulation, craving levels did not change. The results of this study were consistent with those of the previous research that used tDCS on craving (23, 25, 28). 

Many animal studies have shown that anodal stimulation increases neuronal firing and cathodal stimulation results in reversed effects (16). Therefore, based on this evidence and in line with other studies (24), it can be assumed that either an increase in the right or a decrease in the left DLPFC activity or vice-versa can lead to craving reduction; and the present research confirmed this point. However, Mondino et al (29) failed to find a significant difference between active and sham groups in the self-reported number of cigarettes smoked using 10 sessions of tDCS over the right DLPFC during 1 month.

DLPFC is one of the main areas of the prefrontal cortex that controls the ability of determining actions, assessing future consequences of current activities, predicting outcomes and social control (32). Therefore, one possible mechanism by which DLPFC stimulation decreased craving is an increase of social control; in other words, participants became more capable to suppress their urges. Another alternative explanation is that stimulation of the prefrontal cortex stimulated dopaminergic pathways. Specifically, mesolimbic DA projections into striatum are hypothesized to regulate food intake by modulating appetitive motivational processes. Dopamine modulation through cortical stimulation has been shown before with tDCS (21). One important result of this study was that tDCS has therapeutic effects on daily smokers as well as social smokers; it especially has remarkable and strong effects on daily smokers. Daily smokers reported fewer number of cigarette consumption after one month from treatment than social smokers, but when participants received sham stimulation, they did not report a decrease in number of cigarettes. Due to damaging effects of craving on emotional, personal, social, economic, and familial aspects of life in daily smokers, according to their self-report, they were more motivated to quit smoking, which is consistent with previous research (30, 31). According to Colder, Lioyd-Richardson, Flaherty, Hedeker, Segawa, and Flay (33), college smokers demonstrate considerable individual variability in their smoking frequency. In short, side effects of smoking are considerable in daily smokers compared to social smokers. Most of our participant in daily smoker groups had retardation in their academic schedules. Daily smokers have dropped out of school, and this mediating factor has led to higher cigarette smoking in these smokers, on the other hand, it also caused great motivation for them to quit after their treatment compared to social smokers.

## Limitation

This study has some limitations. First, there is a need to replicate these findings with a larger sample size in different groups. The effect of tDCS on female smokers was not investigated in the present study. Thus, further studies should confirm the effects of tDCS on female smokers. Also, one of the measures that was used to assess craving was self-reported questionnaire by the participants. One of the limitations of self-reported questionnaire is inaccuracy. Therefore, future studies should consider other quantitative measures to assess smoking behavior and evaluate its efficacy in addition to standard pharmacological treatments.

## Conclusion

Despite available treatments for tobacco addiction, there are many smokers, particularly those with high dependency who are unable to quit, and the relapse rate is very high. Therefore, new brain stimulation techniques can be useful for them. In line with the above-mentioned points, the results of the present research showed that 10 sessions of tDCS applied over DLPFC reduced craving in daily and social smokers.
